# Expanded Pharmacy Practice Implementation: Lessons from Remote Practice

**DOI:** 10.3390/pharmacy10010015

**Published:** 2022-01-12

**Authors:** Selina Taylor, Alice Cairns, Beverley Glass

**Affiliations:** 1 Centre for Rural and Remote Health, James Cook University, Mount Isa, QLD 4825, Australia; 2 Centre for Rural and Remote Health, James Cook University, Weipa, QLD 4874, Australia; alice.cairns@jcu.edu.au; 3 Pharmacy Department, James Cook University, Townsville, QLD 4825, Australia; beverley.glass@jcu.edu.au

**Keywords:** model of care, extended practice, rural pharmacy, innovation, pharmacy services

## Abstract

Aim: The aim of this study is to explore pharmacist perspectives of the implementation of a community pharmacy-based ear health service in rural communities. Method: A community pharmacy-based health service model was designed and developed to provide an accessible ear care service (LISTEN UP—Locally Integrated Screening and Testing Ear aNd aUral Program) and pharmacist’s perspectives of the implementation of LISTEN UP were explored. Thematic analysis was conducted and data coded according to the Consolidated Framework for Implementation Research. Results: A total of 20 interviews were conducted with 10 pharmacists, averaging 30 min. Visualistion of the ear canal was reported as the greatest advantage of the service, whilst the time required for documentation reported as a complexity. The number of pharmacists working at one time and the availability of a private consultation room were identified as the two limiting factors for execution. On reflection, the need for government funding for service viability and sustainability was highlighted. Discussion/Conclusion: Expanded pharmacy practice is emerging for the Australian pharmacy profession. Rural community pharmacists are recognised as integral members of healthcare teams, providing accessible medication supply and health advice to seven million people in Australia who call rural and remote regions home. However, there are no structured models supporting them to provide expanded services to improve health outcomes in their communities. This study provides lessons learnt to guide future design and development of expanded models of pharmacy practice.

## 1. Introduction

Rural pharmacists are integral members of the healthcare team and are at times the only permanent health professionals in small remote communities [1]. Rural community pharmacists provide accessible healthcare including medication supply, stewardship and safety and are often the first point of call for health advice for seven million rural and remote Australians [1,2]. They are dedicated health professionals who provide pharmaceutical services to local communities, however, the full scope of their practice is not well utilised or understood (ref HP article) [3,4].

Australia’s vast and varying landscape is home to resilient, rural and remote people who are major contributors to Australia’s economy through agricultural and mining industries [5]. A reduced life expectancy, higher rates of disease and injury, and complex health profiles are some of the adversities for rural people, predominately due to the distance from metropolitan healthcare settings [2,5].

Rural pharmacists internationally are trialling an expanded scope of practice to provide innovative, targeted health services to meet the needs of their communities and improve health outcomes [6]. Expanded practice (also known as extended practice) has evolved to include immunisations, screening and management of chronic and infectious diseases (including diabetes, cardiovascular disease, sexually transmitted infections) and health promotion [6,7]. However, in Australia, despite rural community pharmacists’ knowledge and embedded role in their community, pharmacists are unable to access structured or funded models of care to provide these expanded services, aside from a limited schedule of immunisations [1]. This, together with a poorly defined role with ambiguous boundaries of their recognised scope of practice has rural pharmacists challenged to provide appropriate care to these vulnerable and complex populations [4].

In response to this, a community pharmacy-based health service model was developed and piloted to provide an accessible ear care service in two rural towns in Australia [8,9]. The service (LISTEN UP—Locally Integrated Screening and Testing Ear aNd aUral Program), provided a structured protocol for trained pharmacists to conduct ear examinations, which included a brief history, hearing screening, otoscopy and tympanometry assessments with an embedded referral pathway to a general practitioner (GP) if required [8].

The aim of this study is to explore the pharmacist perspectives of the implementation of a community pharmacy-based ear health service in their rural communities.

## 2. Data and Methods

### 2.1. Study Design

A qualitative approach informed by an ethnographic lens of rural culture was used to explore pharmacists’ perspectives of a rural community pharmacy-based ear health service [10]. Rural pharmacists were interviewed using in-depth semi-structured interviews [8]. This is part of a larger mixed-methods health services research project [10].

Implementation science [11], defined as “the scientific study of methods to promote the systematic uptake of research findings and other evidence-based practices into routine practice, and, hence, to improve the quality and effectiveness of health services”, has provided the theoretical framework, specifically the Consolidated Framework for Implementation Research (CFIR) [12], for this study. The CFIR comprises 5major domains, incorporating 39 constructs, relevant to the implementation of a novel intervention or policy [13]. Damschroder (et al. 2009) has detailed a description of each of the constructs, with a rationale and definition [13] (Table 1).

### 2.2. Participants, Setting and Recruitment

During February–July 2021, all of the pharmacists (n = 10) who were participating in the LISTEN UP health service were invited via email to be interviewed [10]. The two trial sites were defined by the geographical classification system Modified Monash Model, as a MMM5 (small rural town) and MMM6 (remote community) [14].

### 2.3. Procedure and Semi-Structured Interview

Participating pharmacists were provided with an information sheet and if they were agreeable to the interview, returned written informed consent. Interviews were audio recorded and de-identified in the transcription process. Interviews were conducted prior to and at completion of the LISTEN UP service. Demographic data including, gender, years of practice, postgraduate qualifications and postcodes were also collected. The schedule of interview questions was informed by previous research in the area and review of the literature [3,4,6,15,16,17,18,19].

### 2.4. Data Analysis

All interview recordings were transcribed verbatim, coded and categorized into emerging themes. Objectivity, assumed knowledge and bias were minimized by engaging six participants in a member checking process to ensure their code/theme interpretations were an accurate representation of their perspective.

The initial conventional content analysis of five transcripts and field notes incorporated both the data-driven inductive approach and the deductive priori template of codes approach [20]. A coding manual containing the initial codes was then developed and those codes that were conceptually related were combined into categories using an ethnographic technique of domain analysis [21]. Analysis was performed with the assistance of software program NVivo 12 [22], using a hybrid approach of inductive and deductive coding [20].

The CFIR was adapted and applied to the analysis [12]. Eighteen constructs derived from CFIR were found in the analysis and termed as codes related to the domains termed as themes (Table 1) [13]. The four constructs relating to the implementation process (planning, engaging, execution, reflection and evaluation) structured the discussion for this study.

### 2.5. Ethics Approval

James Cook University Human Research Ethics Committee granted ethical approval (H8187).

## 3. Results

Interviews were conducted both pre- and post-intervention. In all, twenty interviews were conducted with ten pharmacists, nine of which were female. The length of time they had been practicing varied from one to twenty-four years, with an average of eight years; three pharmacists had completed postgraduate qualifications. The interview duration ranged from 14 to 62 min, averaging 30 min.

Table 1 provides the data categories derived from the thematic analysis with a description of the codes incorporated based on the CFIR [13]. Direct quotations are numbered (PX-Pharmacist) with a non-identifying descriptor included.

### 3.1. Characteristics of the Intervention

Participants described numerous advantages of the LISTEN UP service. Broadly, there was a perception that the service built a rapport with consumers and GPs. Increased trust, returning customers and consumers recognizing the community pharmacy as a health care destination rather than a medication supply store were highlighted.

“It’s one of those things like vaccination services, that changes people’s perception of what a pharmacy can offer and what a pharmacist can do. It stops people thinking that pharmacy is just for your medication.” (P1)

An acknowledgement was made that ear care in current pharmacy practice was not routinely or comprehensively provided and that the visual aid provided by the video-otoscope allowed pharmacists to engage, reassure and educate patients about their ear health.

“I felt as though I was getting a lot more engagement with the patients when I was actually showing them (their ear canal) and being able to show them why I was thinking the way I was thinking in regard to their treatments.” (P4)

The adaptability of the service was highlighted as an enabler. Participants expected that the LISTEN UP model could be adapted and applied to other services, which could be delivered in community pharmacy such as advanced hearing testing, wound care, and the management of minor ailments. Similarly, one of the sites had difficulty making required GP appointments for their patients, however they were able to adapt the service model to provide the referral documents and examination results to telehealth GP service providers to ensure timely access to care.

Some participants lacked confidence in providing the service after the training was completed due to a lack of trialability. Suggestions of increasing the number of case studies provided during the training, having more people to examine during the training, and having feedback from an expert for the first 10–20 examinations were made. For some participants, a lack of prior knowledge about ear health contributed to their lack of confidence and for others there was hesitation and uncertainty about providing a physical service.

“I can tell normal versus abnormal, but the severity of the abnormality, I’m not sure, I think it’s just a function of not looking at a lot of ears. But also, getting some real-time feedback would be great as well, but you’ve got no one to really verify it with.” (P7)

The complexity of the service was also acknowledged, with most of the difficulty being associated with completing the documentation required to deliver the service, which was not integrated into the existing pharmacy software programs. Some of the pharmacists managed this by utilising a pharmacy assistant to complete the documentation and others omitted the documentation process entirely.

“Some pharmacists don’t actually record it when we do it, so we probably are seeing and doing a lot more than what we’ve recorded.” (P2)

Overall participants were happy with the design quality of the service. Positive comments described included consumer acceptance of the service, no charge to consumers, the pre- and post- workshop training components, and ensuring all pharmacists employed in the two pharmacies completed the training. Some participants expressed a desire for more explicit training on the clinical guidelines (including prescribing guidelines) for ear pathologies as well as training specifically targeted for the pharmacy profession. In addition, a more detailed protocol with less decision making was suggested.

“We need better pharmacy-specific stops. What to do, step by step, this is what we do, this is when we refer, this is how it sits, this is where you record it, how you record it.” (P7)

The embedded referral process, whereby pharmacists were able to book same-day or next-day appointments with GPs was also described as an integral component of the model.

“As part of the trial, we can guarantee that today you’ll see a doctor if it is important enough. We’ll see whether we can manage it in the pharmacy. If we can’t do it then you can see your doctor today, which is something I don’t think otherwise we’d be able to offer.” (P3)

Cost of the intervention was a major enabler to service uptake. The service was provided at no cost to the consumers, however, the pharmacists were not remunerated for the time spent providing the service, though all equipment and training was funded. As there was no remuneration for the service, at busy times the service was not prioritised and not offered to consumers. Pharmacists expected that patients would not be willing to pay for the service, unless there was an opportunity to prescribe antibiotics if required.

“If I could offer the same service as a GP, there’d be value in it, because it would be saving them either a trip to hospital emergency department or a $45 out-of-pocket expense for an on-day GP appointment. But if I can’t give them the piece of paper and the antibiotics at the end of it, it’s just not valuable.” (P1)

### 3.2. Inner Setting

Within the inner setting of the pharmacy, participants described a number of structural characteristics that contributed to the implementation and execution of the service. The number of pharmacists working at one time was a limiting factor to service being offered to the consumers. All of the participants agreed that the service was not offered if there was only one pharmacist working, e.g., on a weekend, and some suggested that three pharmacists on duty at any one time were required to be able to provide the service.

“We’re pretty fortunate with our staffing to be able to do it, but if I was here alone, I don’t do it.” (P3)

“It would have affected workflow if there was only one to two pharmacists on, but because we have three or four on, on the daily, it was easy to integrate in.” (P9)

In addition, the availability of a consultation room influenced the pharmacists’ capacity to offer the service. As vaccination services increased with COVID 19 and Influenza, the availability of the LISTEN UP service was reduced.

“We only have one room, so I think if it’s we are busy with flu vaccinations, it makes it really difficult to offer another service…we pretty much, have somebody vaccinating all the time.” (P1)

The number of potential consumers that would be missed, who were served by pharmacy assistants, was also discussed. Some strategies that were employed to minimise this included putting alerts on ear products so when they were scanned on a register, that would flag the LISTEN UP service; however, it was identified that the pharmacy assistants would also require appropriate training about the LISTEN UP service to know when to offer it to consumers.

The importance of the network and communication process between the pharmacy and the GP practice was described as vital to success of the service. Initially, the pharmacists reported difficulties in making GP appointments for consumers within an appropriate timeframe (same-day or next-day), however this was a barrier attributed to the administrative reception staff who were not aware of the service.

“A lot of the times they would say that they didn’t have an appointment available that day or the following day and then I’d speak to the doctor and get an appointment…but it was a lot of going back and forth, and that would take up a lot of my time.” (P10)

All of the participants reported a strong connection to professional pharmacy practice culture. Pharmacist owners reported professional services as a valued aspect of their business model.

“Doing professional services well, shows that we are reliable, we’re trustworthy, we have the knowledge, we have the education and the passion, as well, to do it and to implement it.” (P1)

The implementation climate proved to be a challenge for some pharmacists in management roles. Those with competing priorities, such as dispensary tasks, found it difficult to dedicate the time required to providing the LISTEN UP service including the documentation process.

“Time is the biggest factor. We’re often under the pump with the supply role, so I think clinical service can press you that little bit further. That’s where you need to have an adequately staffed pharmacy to be able to provide clinical services.” (P8)

However, a shift towards dedicating pharmacists to professional services was also recognised, and strategies including utilising pharmacy assistants to support consumers to complete any required documentation and supporting intern pharmacists to deliver the professional service were noted as a means of operationalising the delivery of expanded services.

### 3.3. Outer Setting

Patient needs and lack of resources were mostly linked to access to GPs. Pharmacists agreed that difficulty in getting a timely appointment at a GP was a major problem for their community, resulting in more complex pharmacy presentations. In addition, the costs and distances required to access a GP appointment and very limited bulk-billing services was forcing consumers to attend hospital emergency departments (EDs). Pharmacists expected that the LISTEN UP service would support the redirection of consumers into appropriate healthcare settings.

“People don’t get access to prescription medicines, or routine medicines that they need in a timely manner. So, things generally compound and they have to get to a worse state before they’ll see doctors.” (P4)

The degree to which there is a positive professional relationship (network) between the pharmacy and the local GP practice (cosmopolitan) was reported as an enabler of the service, however, this support might not translate beyond the local relationships in order to enact broader policy or practice change.

“The GPs are really supportive of us. Not so sure about the medical associations, they tend to have a different perspective.” (P9)

Participants did not describe any peer pressure from other pharmacy competitors due to the innovative nature of the service, however they did describe prior anecdotal resistance from the medical professional towards pharmacists providing expanded services.

### 3.4. Individuals Involved

During the engagement phase of the implementation of the service, all of the participants broadly reported positive attitudes (knowledge and beliefs) towards expanded practice and specifically to the LISTEN UP service.

“I’m very passionate about working at an expanded scope, and very passionate about bridging the healthcare gap between rural and metropolitan. I want to do everything I can to make sure that just because we live in a rural area, it doesn’t mean that we’re disadvantaged in terms of our access to healthcare.” (P7)

Self-efficacy of participants widely varied from those who were very confident to provide the service, to those who lacked confidence, and this was closely connected to the number of consultations that those pharmacists had provided. Pharmacists who conducted the majority of the services were quite comfortable with continuing the service into the future.

“I’m still not confident, and I think that’s just purely from not looking at a lot of ears.” (P7)

All of the participants were motivated to work at an expanded scope and to implement the LISTEN UP service. The individual stage of change amongst the participants varied between determination and action, as some pharmacists were already providing expanded services such as vaccinations [23].

“I’ve always been a big advocate for expanded practice. People that are drawn to work in health want to help and want to better themselves and do more research, so yes, we’re perfectly placed.” (P1)

Personal attributes such as a preference for usual pharmacy tasks compared to professional services and the recent implementation of other professional services such as pain or sleep clinics impacted on the pharmacists’ motivation to participate in the LISTEN UP service.

“I probably haven’t been one of the drivers of the trial. There are others in my pharmacy who much prefer it than I do, so I’m usually the one where I’m like, go on, get in there, and I’ll do this stuff out here and keep the pharmacy running.” (P2)

## 4. Discussion

Expanded pharmacy practice globally has shown potential to improve consumer access to healthcare and patient health outcomes [24]. In previous research, consumers and health professionals have reported some resistance to expanded practice, however, when focusing on rural Australia, pharmacists, consumers, health professionals and stakeholders are all supportive of expanded practice for rural and remote areas with an expected improvement in health for their local communities [3,15,16,17,18,24].

The analysis of the implementation of an innovative expanded health service has identified various factors to consider in improving the success of future services. This study has reported pharmacist perspectives of various constructs connected to the CFIR framework of implementation [13]. The lessons learnt from the implementation of LISTEN UP can be applied to the four stages of implementation: planning, engaging, executing and reflecting and evaluating [12].

## 5. Planning

During the planning stage it was recognised that the LISTEN UP service needed to align with the local burden of disease and existing available ear care providers. The pharmacists described ear health to be a problem in their community, which is aligned with national reports of ear disease of rural Australia [25]. In addition, difficulty in accessing GP appointments and needing to travel extensive distances to specialist ear services were proving to be a major barrier for consumers in their community [18]. Reports of people delaying their attendance to GP practices and instead attending hospital emergency departments potentially further impacts on the health of remote populations [2,26]. These issues are likely to worsen and compound when the full impacts of COVID-19 on primary healthcare are realised [27], thus highlighting the need to support rural community pharmacies to play a larger role in primary healthcare, through the provision of expanded services.

Training was also an important consideration during the planning stage of LISTEN UP. Previous research had highlighted the importance of high quality training to ensure that pharmacists were able to provide an expanded service safely and effectively, and this was described by both rural pharmacists and health professionals [3,18]. In addition, a lack of understanding of the level of training pharmacists received made health professionals uncertain about pharmacists’ capability to provide expanded services [4]. Some pharmacists who participated in the trial, although they found the training valuable, still lacked confidence in clinical decision-making and suggested that a more structured protocol that reduced decision making would have improved their confidence providing the service. A similar trial being conducted in Australian pharmacies managing urinary tract infections has a very clear and directed protocol and this model was suggested by the pharmacists [28]. The lack of confidence to make clinical impressions and decisions has been previously reported and suggested to be an area of pharmacy practice that will need to be strengthened for expanded service implementation in the future [4,29,30].

## 6. Engaging

The culture of professional practice is emerging in community pharmacy as the profession evolves from a model of medication supply to a pharmaceutical care model [31]. The pharmacies that participated in this trial both have a progressive approach to pharmaceutical care. This is essential for expanded practice implementation as previously tasks such as preparing dose administration aids and performing blood pressure checks have been considered as an advancement in pharmacy services and for some pharmacies would still be considered expanded practice [18]. However, as we have learnt from our international counterparts, rural community pharmacists have the potential to play a much larger role in healthcare [6].

The collaboration with GPs has been integral to LISTEN UP. The embedded referral pathway to appropriately timed GP appointments has allowed patients to be fast-tracked into an appropriate healthcare setting. Concerns about the level of GP support for expanded practice has been previously identified [32], however, workload pressures on rural GPs has assuaged concerns provided that services are delivered safely [18]. Diverting minor ailments from emergency departments to more appropriate primary health care providers (pharmacies and GP’s) is embedded in health service policies [33]. A third of ED presentations are able to be diverted to GPs or pharmacies and in Queensland alone, 612 ED presentations were for ear wax blockages over a 6 month period [33]. In rural and remote areas, GP appointment wait times can be many weeks, further supporting the need to provide expanded community pharmacy models of care as pharmacists are the most accessible health care professional in many of these communities. [18,34].

## 7. Execution

The execution stage of the LISTEN UP trial identified two limiting factors for the pharmacies to be able to offer the service to consumers: the number of pharmacists working at one time and the availability of a private consultation room. Both the pharmacies were well staffed with pharmacists; however, afterhours and weekends saw single on-duty pharmacists unable to prioritise the LISTEN UP service. It has been identified that community pharmacists place importance on providing professional services, however, competing essential pharmacy activities hamper their ability to dedicate the time to provide those services [35].

In addition, both pharmacies had private consultation rooms, however, one pharmacy had only one room, which was often being utilised and prioritised for vaccination services. These two barriers have been recently identified by other rural health professionals also as the two biggest challenges to pharmacists providing expanded services [18]. Although private consultation rooms reduce retail space, most community pharmacies have constructed a private room to be able to offer vaccination services, however, guidelines about specifications of pharmacy consultation rooms have not been published [36].

## 8. Reflection and Evaluation

On completion of the LISTEN UP trial, all of the participants described the need for government funding for the service to be viable and sustainable for the future. Although consumers reported a willingness to pay for this service and expanded services broadly, it is well recognised that the most vulnerable patients are unlikely to have the ability to pay for the service [10]. In particular, the Australian Indigenous populations are likely to experience financial constraints as a barrier to accessing professional services and given their health outcomes are the worst in the country, a government funded program is needed to remove the additional barrier of cost [37].

## 9. Conclusions

The implementation of expanded services in rural community pharmacies is a staged process which requires many considerations. Examination of pharmacist perspectives of the implementation of the LISTEN UP service has highlighted important lessons which can be applied to the design and development of future expanded pharmacy practice. A targeted service, in which pharmacists are adequately trained to provide services and which has an embedded collaboration with GPs, is essential. Such a service needs to be provided by community pharmacists with a strong culture of professional practice with pharmacist time and private consultation space readily available. Funding is critical for expanded services such as LISTEN UP to be sustainable and this funding should be directed from either government, or other sources, rather than consumers. Expanded practice for rural community pharmacy is a pivotal strategy in reducing the barriers to accessing primary healthcare for rural and remote Australians. Lessons from this remote practice model has the potential to provide valuable insight into the design and development of future models of expanded pharmacy practice.

## Figures and Tables

**Table 1:** Definition of Role Constructs and Examples.

Theme	Codes	Definition	Exemplar
Characteristics of the intervention	Relative advantage	Perception of the advantage of implementing the intervention versus an alternative solution.	“It’s really nice showing them what their eardrum looks like and explaining to some why they don’t need antibiotics.” (P10) “We don’t have existing ear care services, so this model has all the advantages, because it’s actually a model and actually a service.” (P7)
	Adaptability	Degree to which an intervention can be adapted, tailored, refined, or reinvented to meet local needs.	“We are already doing consultations on wound care, and on skin care, people already come and see us for that type of stuff so the LISTEN UP model with the GP referral pathway would be good.” (P9)
	Trialability	Ability to test the intervention in the organisation and to be able to undo the implementation if warranted.	“From a training perspective, we need to see more abnormal photos and the variance. Seeing lots of examples, so you’re more familiar, so you know how to accurately diagnose or even some real-time feedback would be good.” (P7) “I felt like I needed the first five, ten hours of practice, mainly just to get comfortable with, actually, how to talk to patients and look inside the ear at all the techniques. After that, I felt very comfortable.” (P4)
	Complexity	Perceived difficulty of the implementation, reflected by duration, scope, radicalness, disruptiveness, centrality, intricacy, and number of steps for implementation.	“The process and referral have worked really well. Paperwork is always a pain, in general that’s probably the one limiting factor if someone just wants to come in quickly, get something done and go out.” (P7)
	Design quality	Perceived excellence in how the intervention is bundled, presented, and assembled.	“That’s why all our pharmacists need to be trained in this, and that’s with our vaccination service too, we will not have somebody on our team that’s not vaccination trained. We all need to upskill together.” (P1) “We don’t have a central spot to be able to record everything, so the fact that we have to open another document, prepare another thing. No just being able to click on the patient, write the patient notes, observations, referrals, whatever, and close it—it needs to be integrated.” (P3)
	Cost	Cost of the intervention implementation including investment, supply, and opportunity costs.	“We do need some remuneration, because I am spending 15 min in the consult room with the person to say, come back and see me, so I can spend another 15 min with you in two days’ time, which I’m not getting any money for.” (P7)
Inner setting	Structural characteristics	Social architecture, age, maturity, and size of the organisation.	“On the weekends or nights where there’s only one pharmacist there, it’s pretty difficult to facilitate. So that’s the time, when I’ve been there, where we’d be like, can you come back tomorrow?” (P1)
	Networks and communications	Nature and quality of the webs of social networks and communication within the organisation.	“When making appointment for patient, the GP administration staff was the hurdle. The doctors were so on board with it, they loved it.” (P9)
	Culture	Norms, values, and basic assumptions of the organisation.	“We prefer to be doing professional services, that’s what we like doing, none of us actually like being in the dispensary…that’s where they get the kicks from, that’s where you feel like you’ve done a great job, warm and fuzzy.” (P1)
	Implementation climate	Absorptive capacity for change and shared receptivity of involved individuals to an intervention.	“The GPs here are so supportive of pharmacists. A lot of them are really thankful for the role that pharmacists can play in bridging the gap. If there wasn’t a gap them maybe there’d be problems, but the doctors here are really super aware of how much of a gap there can be in rural health.” (P5)
Outer setting	Patient needs and resources	Extent to which the patient needs (including barriers and facilitators to the needs) are known and prioritised by the organisation.	“It’s in a timely manner is the biggest problem. So we may have the services, but you can’t get it quick enough and you just might have to travel for it.” (P2)
	Cosmopolitan	Degree to which an organisation is networked with other external organisations.	“I think the doctors themselves were all on board, and really excited about it, because I think also, they saw that we were valuing their time.” (P5)
	Peer pressure	Mimetic or competitive pressure to implement an intervention.	“I don’t think we’ve really robbed any other health practice from those services. We’re just doing things that probably wouldn’t have been captured, because there’s no appointment necessary.” (P1)
Individuals involved	Knowledge and beliefs about the intervention	Individuals’ attitudes toward and value placed on the intervention.	“It’s actually a really good idea, and a really good step forward. It’ll take the burden off a lot of the GPs as well, and giving people greater access by not having to see their GPs. So, I have, overall, positive thoughts towards rural pharmacists expanding their scope.” (P10)
	Self-efficacy	Individual belief in their own capabilities to execute the course of action to achieve implementation goals.	“Pre-trial confidence with ear complaints would be a three or a four and post-trial let’s say seven, only because I feel, unfortunately, I didn’t get that much practice. Because of working part time, I didn’t get to get so many cases.” (P6)
	Individual stage of change	Characterisation of the phase an individual is in as they progress toward skilled, enthusiastic, and sustained use of the intervention.	“I think expanded practice is very important, given that in rural and remote communities it’s hard to get primary healthcare in the form of a GP appointment. So, for there to be expanded pharmacy services so that people are able to get their healthcare needs met, it’s very important. And that’s why we need expanded scope in the practice, because within our standard scope we wouldn’t be able to help quite a large section of people get their primary healthcare needs.” (P4)
	Other personal attributes	Other personal traits	“I’m actually used to doing sleep apnoea consults, which can take up to 40 min sometimes. And, because we make a solid profit out of it, I don’t feel bad taking that time. So, maybe I’m just more used to that consultative practice.” (P6)

## Data Availability

Data is contained within the article.
